# Anti-Estrogenic Activity of Guajadial Fraction, from Guava Leaves (*Psidium guajava* L.)

**DOI:** 10.3390/molecules25071525

**Published:** 2020-03-27

**Authors:** Jaqueline Moraes Bazioli, Jonas Henrique Costa, Larissa Shiozawa, Ana Lúcia Tasca Gois Ruiz, Mary Ann Foglio, João Ernesto de Carvalho

**Affiliations:** 1 Faculty of Pharmaceutical Sciences, University of Campinas, 13083-859 Campinas, SP, Brazil; jaqueline.bazioli@gmail.com (J.M.B.); lashiozawa@gmail.com (L.S.); ana.ruiz@fcf.unicamp.br (A.L.T.G.R.); maryann.foglio@fcf.unicamp.br (M.A.F.); 2 Institute of Chemistry, University of Campinas, P.O.Box 6154, 13083-970 Campinas, SP, Brazil; j161206@dac.unicamp.br; 3 Postgraduate program in Dentistry, Piracicaba Dental School, University of Campinas, 13 414-903, Piracicaba, SP, Brazil

**Keywords:** *Psidium guajava* L., anti-proliferative activity in vitro, estrogenic/anti-estrogenic activity

## Abstract

The research of natural products has allowed for the discovery of biologically relevant compounds inspired by plant secondary metabolites, which contributes to the development of many chemotherapeutic drugs used in cancer treatment. *Psidium guajava* leaves present a diverse phytochemical composition including flavonoids, phenolics, meroterpenoids, and triterpenes as the major bioactive constituents. Guajadial, a caryophyllene-based meroterpenoid, has been studied for potential anticancer effects tested in tumor cells and animal experimental models. Moreover, guajadial has been reported to have a mechanism of action similar to tamoxifen, suggesting this compound as a promisor phytoestrogen-based therapeutic agent. Herein, the anti-estrogenic action and anti-proliferative activity of guajadial is reported. The enriched guajadial fraction was obtained by sequential chromatographic techniques from the crude *P. guajava* dichloromethane extract showing promising anti-proliferative activity in vitro with selectivity for human breast cancer cell lines MCF-7 and MCF-7 BUS (Total Growth Inhibition = 5.59 and 2.27 µg·mL^−1^, respectively). Furthermore, evaluation of anti-estrogenic activity in vivo was performed demonstrating that guajadial enriched fraction inhibited the proliferative effect of estradiol on the uterus of pre-pubescent rats. These results suggest a relationship between anti-proliferative and anti-estrogenic activity of guajadial, which possibly acts in tumor inhibition through estrogen receptors due to the compounds structural similarity to tamoxifen.

## 1. Introduction

Cancer is one of the leading causes of death worldwide [1], with an estimation of 27 million new individual diagnoses by 2050 [2]. Thus, there is a constant demand for new approaches for cancer therapy to be used instead of the conventional treatments that can cause side effects [1,2]. Natural products have demonstrated a powerful alternative in cancer chemotherapy, providing many structures or inspiring the construction of novel anticancer molecules. Many compounds from plants or microorganisms are reported as anti-cancer agents [3,4]. One of the most known examples are vinblastine and vincristine; compounds obtained from *Catharanthus roseus* (*Vinca rosea*) demonstrating to have potent cytotoxic effects [3].

*Psidium guajava* L. (Myrtaceae), a native species from tropical areas, has been reported as a good source of secondary metabolites with pharmacological activity, such as flavonoids, terpenoids, phenols, tannins [5,6]. Among the medicinal uses *P. guajava* extracts and oils are reported to display anti-inflammatory [7], antibacterial [8], antidiabetic [9], anticough and antidiarrheal activities [10]. The cytotoxic activity, against the most diverse types of cancer has been vastly explored [11,12,13,14,15]. However, further investigation is needed to elucidate the mechanisms by which guava regulates cell cycle and apoptosis after treatment [15].

**Figure 1:**
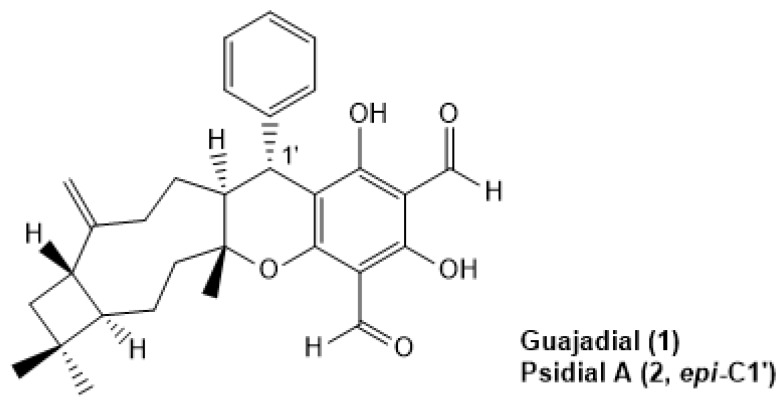
Guajadial and Psidial A structures, meroterpenoids from *Psidium guajava* L. leaves.

Our research group have demonstrated that guava’s dichloromethane leaves extract and the mixture of guajadial (**1**), psidial A (**2**), psiguadial A and B presented significant in vitro anti-proliferative effect against nine human cancer lines [16]. More, this in vitro effect was translated into tumor growth inhibition in Ehrlich’s solid tumor model besides anti-estrogenic activity evidenced by the uterotrophic model where animals were treated through the intraperitoneal route. These results suggested a possible tamoxifen-like mechanism of action for the meroterpens identified in *P. guajava* leaves extract [16]. By using in silico molecular docking simulations, guajadial and psidial A (Figure 1) demonstrated physicochemical properties similar to estradiol and tamoxifen fitting into the estrogen receptors, corroborating that **1** and **2** may act as selective estrogen receptors modulators (SERMs) [16]. Nevertheless, not much information has been reported concerning the role and mechanism of action of guajadial in tumor inhibition or even the influence of route of treatment on in vivo efficiency. In this context, this study aimed to evaluate the anti-proliferative activity and anti-estrogenic action of the guajadial-enriched fraction obtained from the *Psidium guajava* L. leaves extract suggesting a possible relationship between anti-proliferative activity and hormonal action. This correlation was confirmed both by E-screen tests and in vivo tests that assessed uterotrophic activity.

## 2. Material and Methods

### 2.1. Commercial Reagents

Hexane, dichloromethane, methanol, acetic acid, p-anisaldehyde, sulfuric acid, dimethyl sulfoxide, trichloroacetic acid, doxorubicin, and colchicine were commercially purchased from Sigma Aldrich.

### 2.2. General Experimental Procedures

Column chromatography (CC): silica gel 60 (70–325mesh, Merck, Darmstadt, Germany), 2 × 50 cm). TLC (thin-layer chromatography): precoated plates (Merck, Darmstadt, Germany), UV detection, and anisaldehyde solution.

### 2.3. Plant Material

*Psidium guajava* L. were collected at CPQBA–Unicamp (Multidisciplinary Center for Chemical, Biological and Agricultural Researches—Paulínia, SP, Brazil) experimental field. The dried specimen was deposited at Herbarium number 1335 under authorization of Brazilian Genetic Resources (SisGen) (010003/2013-3).

### 2.4. Extraction and Fractionation

*P. guajava* leaves were ground with dry ice and extracted with dichloromethane (1:3, *w/v*) by mechanical stirring (three times, 1.5 h each cycle, renewing the solvent at each step) at room temperature. The partial extracts were combined and the organic solvent was eliminated under vacuum by rotary evaporation system at 40 °C (Büchi Rotavapor RE 120, Flawil, Switzerland). The resulting dichloromethanic crude extract (DCE) was weighted and kept under refrigeration. Then, DCE (35 g) was homogenized with diatomaceous earth Celite^®^ 545 (Merck, Darmstadt, Germany) (1:1, *w/w*) and the mixture was fractionated by adsorption liquid chromatography over Silica gel 60 (Merck, Darmstadt, Germany) 0.0063–0.200 mm thickness. Elutent polarity was increased by gradients of hexane, dichloromethane, and methanol. The 50 mL fractions were collected. Obtained fractions were grouped according to thin-layer chromatography (TLC) profile (visualized with anisaldehyde reagent (acetic acid, *p*-anisaldehyde, sulfuric acid (48:1:1)) followed by heating at 110 °C, resulting in three fractions named A, B, and C (Figure S1). Fraction B (14 g) was further fractionated by flash column chromatography over Silica gel 60 (Merck, Darmstadt, Germany 0.04–0.063 mm thickness) with an isocratic system of dichloromethane and 0.3% methanol. 20 mL fraction were collected. Five fractions were obtained after grouping by TLC profile and named B1, B2, B3, B4, B5 (Figure S2).

B3 fraction (1.6 g) was fractionated on reverse phase Phenomenex (Torrance, CA, USA) column C-18 (55 µM, 70 A, 5 g/20 mL) with eluting mobile phase of deionized water, acetonitrile/water mixture (95:5, *v/v*), and methanol. The 2 mL fractions were collected, resulting in five fractions (B3.1, B3.2, B3.3, B3.4, and B3.5) after grouping by TLC profile (Figure S3).

Fractions B and B3 were selected for further fractionation after gas chromatography–mass spectrometry (GC-MS) analysis indicating the presence of compound **1**. Resulting fraction B3.2 was solid (600 mg) and was named F_FINAL_ since was chemically characterized by GC-MS and contained the active compounds **1** and **2**. Thus, F_FINAL_ was tested by in vitro and in vivo assays.

### 2.5. Chromatographic Analysis

The GC/MS analysis was carried out using Agilent (Palo Alto, CA, USA) gas chromatograph HP-6890/5975 system equipped with an HP-5 (30 m × 0.25 mm × 0.25 μm). Temperature program: 60 °C (3 °C·min^−1^)–240 °C (20 min), injector 220 °C, detector 250 °C. Helium was used as a carrier gas (0.7 bar, 1 mL·min^−1^). The MS were taken at 70 eV. Sample volume was 1–2 μL. The mass spectra were compared with software NIST05 MS Search.

### 2.6. In Vitro Pharmacological Evaluation

#### 2.6.1. Cell Lines and Culture Conditions

Human tumor and immortalized cell lines (Table 1) were grown in complete medium Roswell Park Memorial Institute (RPMI) medium 1640 (GIBCO, NY, USA) containing 5% Fetal Bovine serum (FBS) (GIBCO, NY, USA) and 1% (*v/v*) penicillin: streptomycin (Nutricell, 1000 U/mL:1000 mg/mL) in a humidified atmosphere with 5% CO_2_, at 37 °C. For the experiments, cell lines were used between passages 4 to 12.

#### 2.6.2. Sample Dilution

The DCE and resulting fractions (5 mg) were initially diluted in dimethyl sulfoxide (DMSO) (50 µL) followed by the addition of complete medium (950 µL). Final concentrations (0.25, 0.3, 0.6, 1.25, 2.5, 5, 25, 50, 100, and 250 µg/mL) were obtained by serial dilution in complete medium, except for E-screen assay. For E-screen experiment, it was used a low estrogen medium (medium RPMI 1640 without phenol red (GIBCO, NY, USA) supplemented with 5% Dextran-charcoal-treated fetal bovine serum (DC-FBS, Hyclone, Logan, UT), and 1% (*v/v*) penicillin:streptomycin (Nutricell, 1000 U/mL:1000 mg/mL)). Diluted as described for samples, Doxorubicin (0.025, 0.25, 2.5 and 25 µg/mL, final concentration) was used as positive control on anti-proliferative assay while Colchicine (0.25 nM, final concentration) was the positive control on cell cycle assay.

#### 2.6.3. Anti-Proliferative Assay

Cells in 96-well plates (Costar, Corning, NY, USA) (100 µL well^−1^, density of inoculation see Table 1) were exposed to dichloromethanic crude extract (DCE) and resulting fractions at 37 °C, 5% of CO_2_ in air for 48 h. Final DMSO concentration (>0.25%) did not affect cell viability. Before (T0) and after (T1) treatment, cells were fixed with trichloroacetic acid (50%, 50 µL well^-1^) and cell proliferation determined by spectrophotometric quantification at 540 nm using sulforhodamine B dye [17]. Using the concentration–response curve for each cell line, the Total Growth Inhibition (TGI, concentration that produces total growth inhibition or cytostatic effect) was determined by non-linear regression analysis using software ORIGIN 7.5^®^ (OriginLab Corporation) [18].

For comparative evaluation among resulting fractions of DE, the criteria described by Fouche et al. (2008) [19] (inactive (TGI > 50 μg·mL^−1^), weak activity (15 μg·mL^−1^ < TGI < 50 μg·mL^−1^), moderate activity (6.25 μg·mL^−1^ < TGI < 15 μg·mL^−1^), and potent activity (TGI < 6.25 μg·mL^−1^)) was adopted to select the most active fraction to be evaluated in further in vitro and in vivo experiments.

#### 2.6.4. E-Screen Assay

MCF7 BUS cells (d.i.: 1 × 10^4^ cell mL^−1^) in 12-well plates were incubated with low estrogen medium (2 mL well^−1^) for 72 h. After that, cells were treated with DCE (5 and 25 μg·mL^−1^) or F_FINAL_ fraction (0.3, 0.6, 1.25 and 2.5 μg·mL^−1^) in absence or presence of 17-β-Estradiol (E2, 10^−9^ M) and incubated for 144 h. Cell viability were determined by spectrophotometric quantification (540 nm) of cellular protein content using sulforhodamine B (SRB) assay, as described for anti-proliferative assay.

#### 2.6.5. Cell Cycle Analysis

MCF7 BUS cells (6 × 10^4^ cell mL^−1^ for 24 h incubation and 4 × 10^4^ cell mL^−1^ for 48 h incubation, 1 mL·mL^−1^) were seeded on 12-well microplates and, after 24 h, were submitted to cell cycle synchronization by FBS suppression. After more 24 h, cells were treated with F_FINAL_ fraction (2.5 and 5 µg·mL^−1^) and incubated at 37 °C, 5% CO_2_ for 24 and 48 h. Colchicine (0.25 μM) was used as positive control. Afterward, cells were trypsinized (trypsin-EDTA 0.25%), washed with Phosphate-buffered saline (PBS) and resuspended in 500 µL of cold ethanol 70%. After 12 h at 4 °C, cells were washed with PBS and resuspended in 200 µL of Guava cell cycle 4500-0220 (Merck/Millipore/Guava Hayward, CA, USA) for 30 min at room temperature and in the dark. Data acquisition (5.000 events per replicate) and analysis were performed using FACS- Canto II cytometer (BD Biosciences^®^, CA, USA), at the Central Laboratory of High Performance Technologies (LaCTAD)/UNICAMP.

### 2.7. In Vivo Assays

#### 2.7.1. Animals

Swiss female mice (fifteen animals) and Balb-C female mice (seventy-three animals), aged 8–10 weeks and weighing 20–35 g, and Wistar female rats, aged 21 days, were used for in vivo assays. The animals were obtained from the Multidisciplinary Center for Biological Investigation on Laboratory Animal Science (CEMIB/UNICAMP) after experimental protocols been approved by the institutional Committee for Ethics in Animal Research, at the University of Campinas (CEUA, UNICAMP 3189-1/2013, 3028-1/2013, 3565-1/2014, 3571-1/2015). All procedures were in accordance with the principles and guidelines adopted by Brazilian College of Animal Experimentation (COBEA).

Animals were housed (5 mice or rats per cage) in polyethylene boxes (matte white) with disinfected softwood beddings, at room temperature of 22 ± 2 °C; relative humidity of 50 ± 20% and light/dark cycle of 12 h/12 h, with free access to food (Nuvilab^®^, Curitiba, Brazil) and potable water.

##### Samples and Drugs Preparation

F_FINAL_ fraction and drugs were emulsified in Tween 80^®^ (Sigma-Aldrich, Saint Louis, MO, USA) (final concentration 0.3%) and dissolved in PBS (pH 7.0). Vehicle was PBS, pH 7.0 + Tween 80^®^ (Sigma-Aldrich, Saint Louis, MO, USA) 0.3%.

### 2.8. Acute oral Toxicity

Fifteen Swiss female mice were fasted for 4 h and then treated orally with F_FINAL_ fraction (100 and 200 mg/kg, *n* = 5/group) or vehicle (10 mL/kg, control group). All animals were observed during 4 h and then daily for 14 days, for general toxicity signals evaluation: bodyweight evolution, locomotion, behaviour (agitation, lethargy), respiration, salivation, tearing eyes, cyanosis, and mortality. At the 14th experimental day, animals were euthanized by deepening anesthesia with a mixture of ketamine (Dopalen^®^ 300 mg/kg, i.p.) and xylazine (Anasedan^®^ 30 mg/kg, i.p.) followed by cervical dislocation [20].

### 2.9. In Vivo Anti-Cancer Evaluation

#### 2.9.1. Ehrlich Solid Tumor

Ehrlich tumor cells were maintained in the ascitic form, until 20 passages, by peritoneal passages in Swiss male mice by weekly transplantation of 5 × 10^5^ tumor cells/animal in PBS (at least, two animals per week). For testing, cells were prepared at a density of 5 × 10^6^ cells/50 µL/animal in PBS after cell viability evaluation with trypan blue in Neubauer chamber.

Ehrlich cells suspension (5 × 10^6^ cells/50 µL/animal) was inoculated subcutaneously in the flank of Balb/C female mice (*n* = 8 animals/group). On the 3rd day, animals (40 mice) were randomly distributed into negative (vehicle, 10 mL/kg, orally (p.o.)), positive (doxorubicin, Europharma, Sao Paulo, Brazil, 3 mg/kg, intraperitoneal injection (i.p.)) and experimental (F_FINAL_, 12.5, 25 and 50 mg·kg^−1^, p.o.) groups. One group, named Satellite (*n* = 8 animals/group), were not inoculated with Ehrlich cells suspension neither treated with any sample. Animals on vehicle and experimental groups were treated orally alternately and those of doxorubicin group were treated intraperitonially every three days during the 12 days. Clinical parameters (locomotion, behavior, pelage aspect and cyanosis) were evaluated every treatment day and bodyweight was recorded at days 0 and 16th experimental day. On 16th day after anesthesia, blood samples (300 µL, in microtubes containing EDTA 10%, 1 drop/tube) were collected from each animal by the retrorbital plexus for blood counting (pocH-100iV Diff, Sysmex, Nordesblabla, Germany). Then, animals were euthanized by deepening anesthesia with a mixture of ketamine (Dopalen^®^ 300 mg/kg, i.p.) and xylazine (Anasedan^®^ 30mg/kg, i.p.) followed by cervical dislocation. Necropsies were performed on each mouse to assess gross toxicity to some organs (kidneys, liver, heart, uterus. and ovaries) and to remove the tumor mass. The relative tumor weight (RTW) was calculated as tumor weight divided by final corporal weight. The growth inhibition ratio was calculated as ((mean RTW_negative control_ − mean RTW_experimental group_)/mean RTW_negative control_) × 100 [21].

#### 2.9.2. Hollow Fiber Assay

Confluent MCF7 cells were harvested with tripsin/EDTA and suspended in working medium (RPMI 1640 supplemented with 10% fetal bovine serum (FBS), (GIBCO, NY, USA) and 1% (*v/v*) penicillin:streptomycin (1000 U/mL:1000 mg/mL)), at 1 × 10^5^ cell/160 μL density. The cells were then gently infused into sterile conditioned polyvinylidene fluoride hollow fibers (Spectrum Laboratories) (MW exclusion = 500 kDa, 1-mm internal diameter), the fibers were heat-sealed and cut at 2.0 cm intervals. Prior implantation, the fibers were incubated for 24 h in working medium in a humidified atmosphere with 5% CO_2_, at 37 °C. After that, the fibers were randomly separated into groups. Six fibers fragments were transferred to 6-well plate (1 fragment per well) containing Thiazolyl Blue Tetrazolium Bromide (MTT) solution (0.55 mg/mL, 1 mL/well). After 4 h-incubation, the absorbance (550 nm) was measured after formazan dilution with DMSO (250 µL/well) to establish the basal value (D0).

The second group of fiber fragments were transplanted (*n* = three fibers/animal) into the peritoneal cavity of immunocompetent Balb-C female mice (*n* = 5 animals/group) under anesthesia (ketamine 0.016 mg/kg + xylazine 0.08 mg/kg, 5 mL/kg). At 3rd day, animals (25 mice) were randomly distributed into five experimental groups that were treated during seven days with vehicle (negative control, 10 mL/kg, via oral (v.o.), daily), doxorubicin (positive control, 3 mg/kg, i.p., every 3 days) and F_FINAL_ fraction (12.5, 25 and 50 mg/kg, v.o., daily). At 8th day (D8), animals were euthanized by deepening anesthesia with a mixture of ketamine (Dopalen^®^ 300 mg/kg, i.p.) and xylazine (Anasedan^®^ 30 mg/kg, i.p.) followed by cervical dislocation. Necropsies were performed on each mouse to assess gross toxicity to major organs. Then the fibers were retrieved, placed into Petri dishes containing working medium and kept at 37 °C for 30 min. After that, fibers were transferred for a Petri dishes containing MTT (1.25 mg/mL) in working medium, incubated for 4 h in a humidified atmosphere with 5% CO_2_, at 37 °C, and transferred to Petri dishes containing protamine sulfate solution 2.5% (24 h, at 4 °C). To spectrophotometric evaluation, fibers were cleaned, air-dried (24 h) and cut in half before formazan extraction (4 h, at 37 °C) with DMSO (250 µL/fiber). Aliquots (100 µL/well) were transferred into 96-well plate and optical density (O.D.) were measured at 550 nm. Results were expressed for each group as ((average O.D._D8_ − average O.D._D0_)/average O.D._D0_) × 100 [22,23,24].

### 2.10. In Vivo Anti-Estrogenic Evaluation

#### Uterothropic Assay

Forty Wistar female rats (21–25 days) were randomly distributed into 5 experimental groups (*n* = 8 animals/group). Positive and negative control groups were orally treated with 17β-estradiol (0.3 mg/kg) and vehicle (5 mL/kg), respectively. The other three groups were treated with 17β-estradiol (0.3 mg/kg, p.o.) followed by F_FINAL_ fraction (12.5, 25 and 50 mg/kg, v.o., daily) treatment for 3 days. Bodyweight was recorded at days 0 and 4th experimental days. On the 4th day, animals were euthanized by deepening anesthesia followed by cervical dislocation and uteri and ovaries were rapidly collected, stripped of adipose tissue and weighted for calculating uterine and ovary relative weight [25,26].

### 2.11. Statistical Analysis

All data were obtained from single experiments with triplicate determinations. Anti-proliferative screening was reported as TGI. Statistical analysis was performed by One-way non-parametric Kruskal–Wallis’s test followed by Dunn’s test, One-way analysis of variance (ANOVA) followed by Tukey’s test or Two-way ANOVA followed by Bonferroni test, depending on the most appropriate test in each case. The tests are described in the results. Statistical analyses were performed with the GraphPad Prism 5 software (GraphPad Software, San Diego, CA, USA).

## 3. Results

### 3.1. Chromatographic Analysis of F_FINAL_

Chromatographic analysis of F_FINAL_ showed the presence of three major compounds (retention time (RT): 28, 45 and 46 min) (Figure S4A). The normalized percentage areas (relative percentage) of the major peaks are presented in Table 2. Fragmentation profile of both peaks (RT 45.01 and 46.71) are very similar, indicating diastereomers compounds.

Gas chromatography was the most appropriate analytical technique for the purpose of comparison with data available in the literature [27]. The comparison of tandem mass spectrometry (MS/MS) spectra for the peaks at RT 45.01 and 46.71 min, with reported data by Yang et al. [27], allows the confirmation of fragmentation pattern for **1** and **2**, isomeric natural products isolated from guava leaves [28], showing major fragments ion peaks at *m/z* 474, 446, 405, 391, 309, 271, and 241 (Figure S4B,C). The peak at RT 28.08 min is due phthalate contamination in solvent. Therefore, the relative percentage of guajadial and its epimer in F_FINAL_ is 100.

### 3.2. In Vitro Assays

#### 3.2.1. In Vitro Anti-Proliferative Screening

DCE showed weak anti-proliferative activity against ovary (TGI = 27.23 μg·mL^−1^, OVCAR-3) and prostate (TGI = 39.94 μg·mL^−1^, PC-3) tumor cell lines. After successive fractionation, fraction F_FINAL_ showed potent activity (TGI values ≤ 6.25 μg·mL^−1^) against ovary (TGI = 2.66 μg·mL^−1^, OVCAR-3), lung (TGI = 2.79 μg·mL^−1^, NCI-H460), prostate (TGI = 3.70 μg·mL^−1^, PC-3), glioma (TGI = 3.90 μg·mL^−1^, U251), breast (TGI = 5.59 μg·mL^−1^, MCF-7) and multi-resistant ovary (TGI = 6.19 μg·mL^−1^, NCI/ADR-RES) tumor cell lines (Table 3 (Figure S5)).

Then, F_FINAL_ was selected for further in vitro anti-proliferative assay against a panel of human breast tumor cells and non-tumor cells (MCF-7 (estrogen-dependent mammary adenocarcinoma), MCF-7 BUS (mammary adenocarcinoma overexpressing estrogen receptor), MDA-MB 231 (negative triple mammary adenocarcinoma) and MCF-710A (non-tumoral breast cells)). F_FINAL_ showed potent anti-proliferative activity against MCF-7 BUS (TGI = 2.27 µg·mL^−1^) and MDA MB 231 (TGI = 5.13 µg·mL^−1^) cells (Table 4 (Figure S6)). The choice of MCF7 BUS cell line to study mechanism of action was based on the higher sensitivity of this cell line to F_FINAL_ treatment, as previously studied by Rizzo et al. [16], which demonstrated that guajadial and psidial A enriched fraction presented uterotrophic activity in the Ehrlich carcinoma solid tumor model, structural similarities with estrogen and possible affinity of the active principles to the estrogen receptors due to its structure. Therefore, MCF7 BUS was chosen for cytometry tests (cell death mechanism and cell cycle).

Breast cell lines: MCF-7 (express the estrogen and progesterone receptor); MCF-7 BUS (estradiol receptor overexpressing); MDA MB 231 (estrogen, progesterone and HER2 negatives); MCF 10A (non-carcinogenic cell line). TGI: Total Growth Inhibition.

#### 3.2.2. E-Screen Assay in MCF-7 BUS Cells

This quantitative test compares cell growth of 17β-estradiol-treated MCF-7 BUS cell line and cultures treated with different concentrations of the F_FINAL_, evaluating the estrogenic activity based on the effects of F_FINAL_ in the presence and absence of 17β-estradiol (E2, 1 × 10^−9^ M). F_FINAL_ concentrations were determined based on TGI values for MCF7 BUS cell line (Table 4). MCF7 BUS cell proliferation increased 2.5 times (*p* < 0.001) after treatment with 17β-estradiol (positive control) comparing to the negative control (cells and medium), indicating the test was valid.

**Figure 2:**
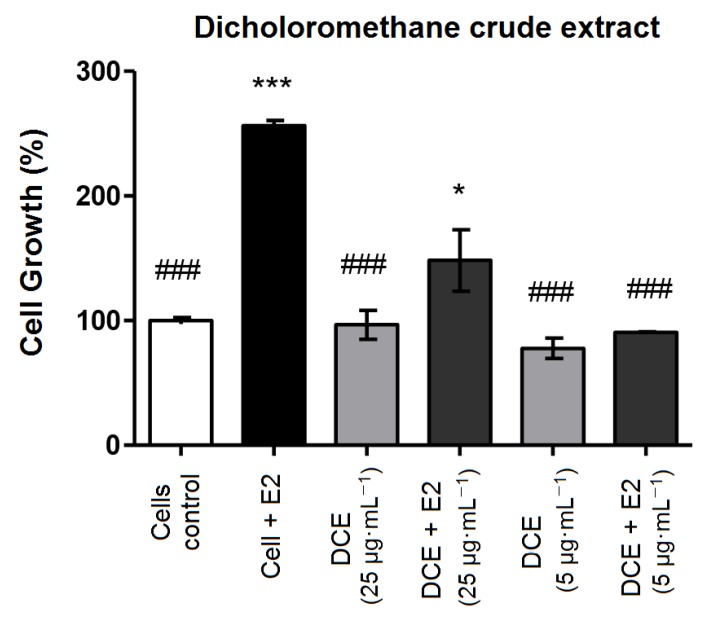
MCF-7 BUS cell proliferation, after DCE treatment in the presence and absence of 17β-estradiol (E2, 10^−9^ M). MCF-7 BUS cells were treated with DCE at concentrations 5.0 and 25 μg·mL^−1^ for 144 h. *, # *p* < 0.05 and ***, ### *p* < 0.001 (analysis of variance (ANOVA)). * Statistical difference compared to control of cells without estradiol. # Statistical difference compared to control of cells with estradiol. Results expressed as mean ± standard deviation. Statistical analysis by One-way ANOVA followed by Tukey’s Test *, # *p* < 0.05 and ***, ### *p* < 0.001 (ANOVA). * Statistical difference in relation to control of cells without estradiol. # statistical difference compared to cells controlled with estradiol.

DCE inhibited cell proliferation of E2 with inverse relationship to concentration (Figure 2), i.e., at the lowest concentration 5 µg·mL^−1^ DCE totally inhibited estrogen action, while DCE-treated cells at 25 µg·mL^−1^ presented a limited increasing proliferation when stimulated by E2. This inversely proportional effect may indicate that DCE act as a partial estrogen receptor agonist.

**Figure 3:**
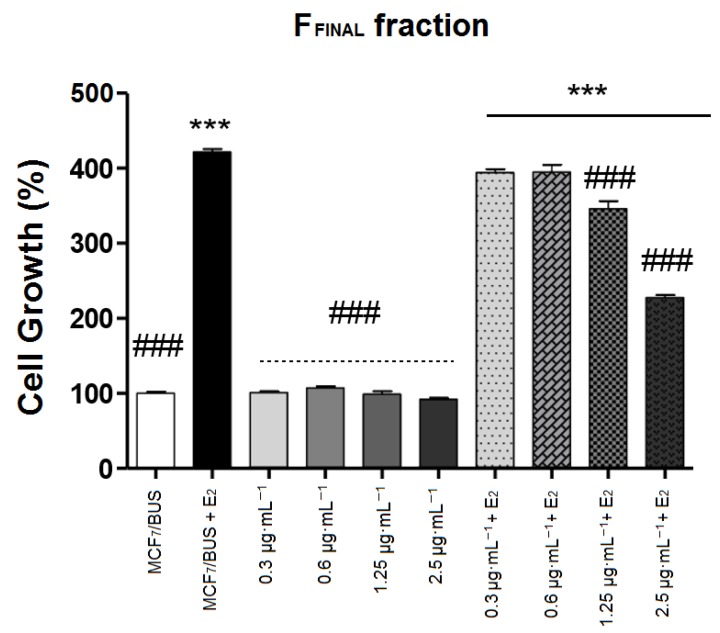
MCF-7 BUS cells treated for 144 h with the F_FINAL_ fraction at concentrations 0.3; 0.6; 1.25 and 2.5 μg·mL^−1^. Results expressed as mean ± standard deviation. Statistical analysis by One-way ANOVA followed by Tukey’s Test ***, ### *p* < 0.001 (ANOVA). * Statistical difference in relation to control of cells without estradiol. # statistical difference compared to cells control with estradiol.

F_FINAL_ fraction at different concentrations (0.3, 0.6, 1.25 and 2.5 μg·mL^−1^) were evaluated (Figure 3). In the absence of E2, F_FINAL_-treated MCF7 BUS cells exhibited similar growth to the untreated cells, regardless of concentration evaluated. Differently, in the presence of E2, the two highest concentrations (1.25 and 2.5 μg·mL^−1^) of F_FINAL_ significantly inhibited the proliferative effect of E2, indicating a possible anti-estrogenic activity.

#### 3.2.3. MCF-7 BUS Cell Cycle by Flow Cytometry

Flow cytometry evaluates cells proportion in a population on several cell cycle stages representing a valuable tool to study mechanisms of action of substances with anticancer potential [29].

**Figure 4:**
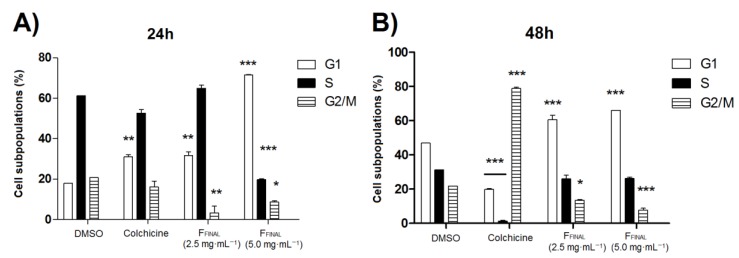
Cell population, expressed in percentage for each phase of cell cycle G1, S and G2, after (**A**) 24 h-treatment and (**B**) 48 h-treatment with vehicle, colchicine (0.25 μg·mL^−1^) or FFINAL fraction (2.5 and 5.0 μg·mL^−1^). Data are derived from an experiment performed; standard deviations are indicated by error bars. Results expressed as mean ± standard deviation. Statistical analysis by One-way ANOVA followed by Tukey’s Test * *p* < 0.05, ** *p* < 0.01 and *** *p* < 0.001 (ANOVA). * Statistical difference compared to cells control.

Therefore, the activity of F_FINAL_ fraction on the MCF-7 BUS cell cycle was evaluated with two concentrations (2.5 and 5.0 µg·mL^−1^) during 24 and 48 h of treatment (duplicate in each treatment). Colchicine (0.25 μg·mL^−1^) was employed as positive control and caused an increase in G1 MCF-7 BUS cell population after 24 h of treatment (Figure 4A). F_FINAL_ fraction (5 µg·mL^−1^) varied MCF-7 BUS cell population after 24 h of treatment, increasing significantly (*p* < 0.001) G1 phase of treated cell (G1—71.54%) compared to cell control (G1—18.06%). Furthermore, a significant reduction (*p* < 0.001) of the populations in both S phase (19.7%) and G2/M phase (8.6%) (*p* < 0.05) related to the cell control group (61.2%—S and 20.69%—G2/M) was observed. The treatment of MCF-7 BUS cells with F_FINAL_ fraction (2.5 µg·mL^−1^) significantly interfered (*p* < 0.01) in G1 and G2/M phases, with a similar profile to cell control only in S phase of the cycle.

F_FINAL_ fraction concentrations at 2.5 and 5.0 µg·mL^−1^ significantly increased (*p* < 0.001) G1 phase cell population (60.5 and 65.9%, respectively) after 48 h, when compared to the cell control group (46.9%) (Figure 4B). Colchicine caused a significant decrease in G1 (colchicine—19.8% and negative control DMSO—46.9%) and S (colchicine—1.3% and negative control DMSO—31.1%) along with a statistically significant increase in G2/M phase (colchicine—78.7% and negative control DMSO—21.9%) of cell cycle, after 48 h.

The F_FINAL_ fraction concentrations of 2.5 µg·mL^−1^ and 5.0 µg·mL^−1^ (Figure 4), promoted a significant increase in G1-phase cell populations after 24 and 48 h, therefore indicating a possible block of cells to proceed to S-phase. Cell cycle progression in G1-phase is regulated by checkpoints that restrict cells with damaged DNA from entering S-phase until damage and high-risk factors are repaired, otherwise, cause cell death or senescence. Stopping the cell cycle allows the repair of this damage by passing the intact genome to each daughter cell [30]. The tamoxifen mechanism of action in cancer cells is well-known for inhibiting DNA synthesis, inducing apoptosis and blocking cell cycle in G1-phase, acting through the inhibition of cell proliferation [31]. Similarly, F_FINAL_ exhibited tamoxifen-like activity and was able to act on the G1-phase of MCF-7 BUS cell cycle.

### 3.3. In Vivo Assays

#### 3.3.1. Oral Acute Toxicity

To evaluate whether in vivo experimental models would reproduce the in vitro anti-proliferative activity observed for F_FINAL_, first the maximum tolerable dose of F_FINAL_ fraction was determined by oral acute toxicity test (single dose).

During the first 4 h after treatment, both 200 and 100 mg·kg^−1^ of F_FINAL_ fraction-induced piloerection, abdominal writhing and lethargy. The most intense signs of toxicity were observed in animals treated with the highest dose than those receiving the lowest dose. All animals recovered after 24 h, with no deaths. Furthermore, no significant difference on the relative organs weight (control and treatment groups) were observed. Therefore, 50 mg·kg^−1^ dose of F_FINAL_ fraction were considered safe as the maximum dose in Ehrlich solid tumor and Hollow Fiber models.

#### 3.3.2. Ehrlich Solid Tumor (Back)

In vivo studies were performed with Ehrlich solid tumor on Swiss mice. The anticancer activity of F_FINAL_ was evaluated in flank tumor models induced by injection of Ehrlich murine tumor cells. The purpose of this test was to evaluate whether oral samples would reduce the development of solid Ehrlich tumor [32].

**Figure 5:**
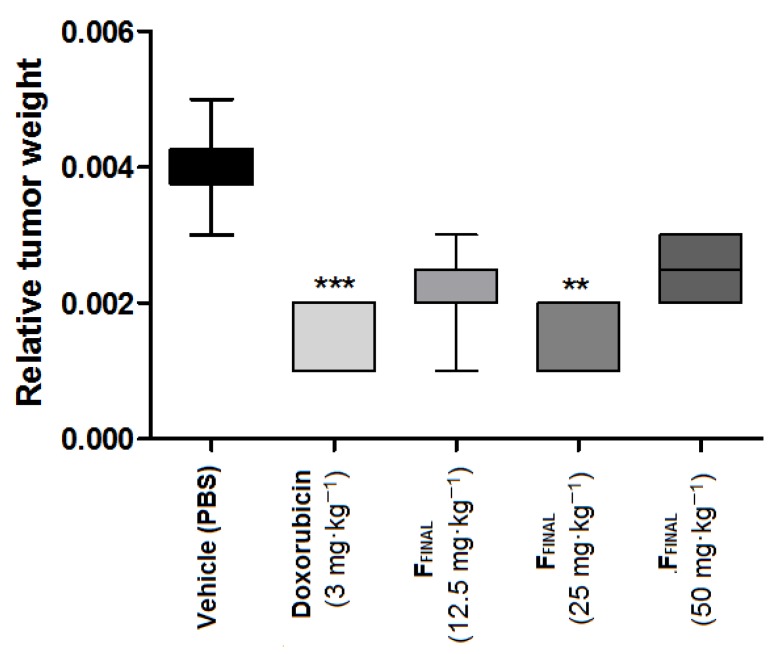
Relative weight, of Ehrlich solid tumor on the flank—F_FINAL_ fraction. Results expressed as mean ± standard deviation; Female Balb/c mice (*n* = 8/group); Treatments (orally every day): vehicle (PBS pH 7.0 + Tween 80, 10 mL·kg^−1^), F_FINAL_ (12.5, 25 and 50 mg·kg^−1^); Treatments (i.p. every three days): Doxorubicin (3 mg·kg^−1^). Statistical analysis by Kruskal–Wallis test followed by Dunn’s Multiple Comparison Test ** *p* < 0,01 and *** *p* < 0,001 compared to vehicle group.

The animals were euthanized on the fifteenth day and average tumor weight of each group was calculated. In vehicle group, tumor weight was 0.082 ± 0.032 g, while F_FINAL_ fraction-treated groups showed mean tumor weight of 0.045 ± 0.007 g, 0.041 ± 0.009 g and 0.058 ± 0.013 g at 12.5, 25 and 50 mg·kg^−1^, respectively. The average tumor weight of doxorubicin control group was 0.027 ± 0.01 g. Considering the relative tumor weight (tumor mass/animal mass), F_FINAL_ fraction oral treatment significantly (*p* < 0.001) inhibited tumor growth, dose-independently, as well as doxorubicin (*p* < 0.001) comparing to the vehicle group (Figure 5).

**Figure 6:**
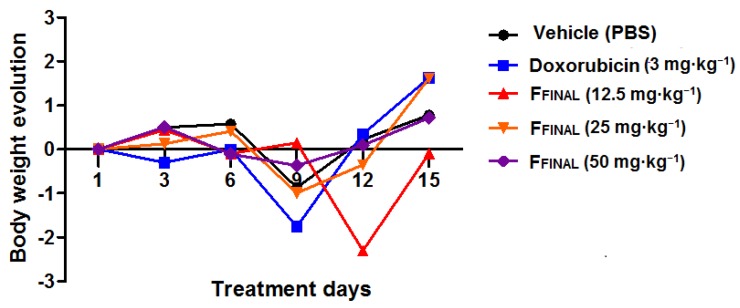
Animal bodyweight variation in Ehrlich solid tumor experiment - F_FINAL_ fraction Animal bodyweight variation during 15 days of treatment; Female Balb/c mice (*n* = 8/group); Treatments (orally every day): vehicle (PBS pH 7.0 + Tween 80, 10 mL·kg^−1^), F_FINAL_ (12.5, 25 and 50 mg·kg^−1^); Treatments (i.p. every three days): Doxorubicin (3 mg·kg^−1^). Statistical analysis by Two-way ANOVA followed by Bonferroni test (*p* > 0.05 for all treatments in comparison to vehicle group).

During the experiment, the animals treated with 25 and 50 mg·kg^−1^ doses of F_FINAL_, as well as those treated with Doxorubicin on the last days (11th to 15th) of experiment, presented clinical signs of toxicity. These signs included piloerection, secretion of tears and pain, along bodyweight loss (Figure 6), resulting in death of one animal from each F_FINAL_-treated groups (50 and 25 mg·kg^−1^). Therefore, the animals of these groups (Doxorubicin, 50 and 25 mg·kg^−1^ of F_FINAL_) did not receive treatment until last day of the experiment (19th day). The interruption in induced bodyweight loss suggesting reversibility of toxicity signs. More, none animal treated with 12.5 mg·kg^−1^ of F_FINAL_ fraction showed any toxicity signs.

**Figure 7:**
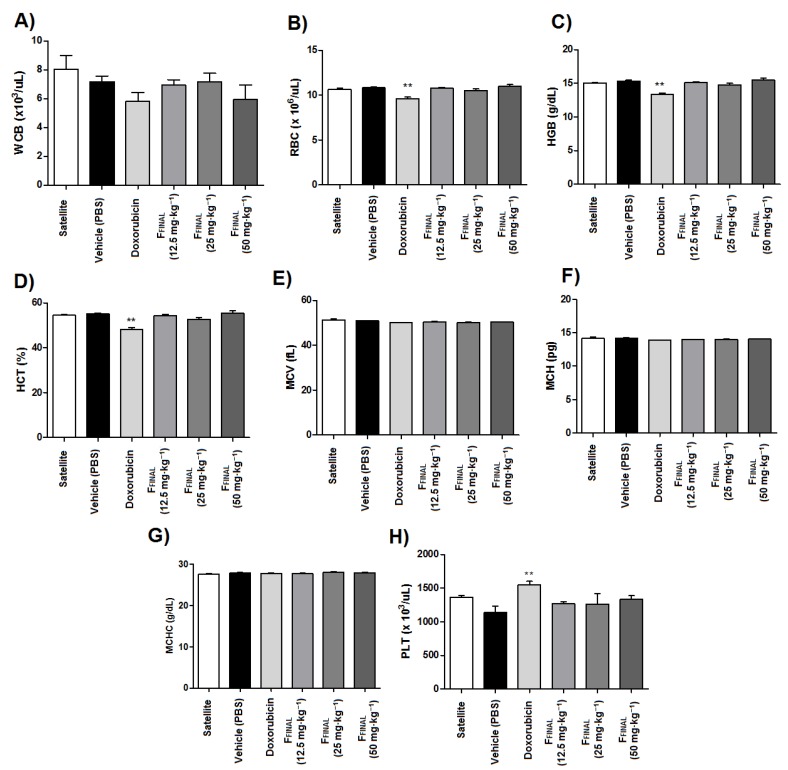
Blood cell count of animals from Ehrlich flank solid tumor experiment—F_FINAL_ fraction. Results expressed as mean ± standard error; Female Balb/c mice (*n* = 8 animals/group); Treatments (orally every day): vehicle (PBS pH 7.0 + Tween 80, 10 mL·kg^−1^), F_FINAL_ (12.5, 25 and 50 mg·kg^−1^); Treatments (i.p., every three days): Doxorubicin (3 mg·kg^−1^); (**A**) total leukocytes (WBC), (**B**) erythrocytes (RBC), (**C**) hemoglobin (Hbg), (**D**) hematocrit (Hct), (**E**) MCV (mean corpuscular volume), (**F**) MCH (mean corpuscular hemoglobin), (**G**) MCHC (mean corpuscular hemoglobin) and (**H**) platelets (PLT). Statistical analysis by Kruskal–Wallis test followed by Dunn’s Multiple Comparison Test ** *p* < 0,01 compared to vehicle group.

The total erythrocyte counts as well as hemoglobin, hematocrit and mean corpuscular volume values were altered in the doxorubicin-treated animals among hematological parameters studied at the end of the experiment (Figure 7). These results confirm the myelosuppression resulting from the administration of chemotherapy. However, no significant effects on blood parameters were observed in animals treated with F_FINAL_ fraction, despite clinical signs of toxicity of this group.

**Figure 8:**
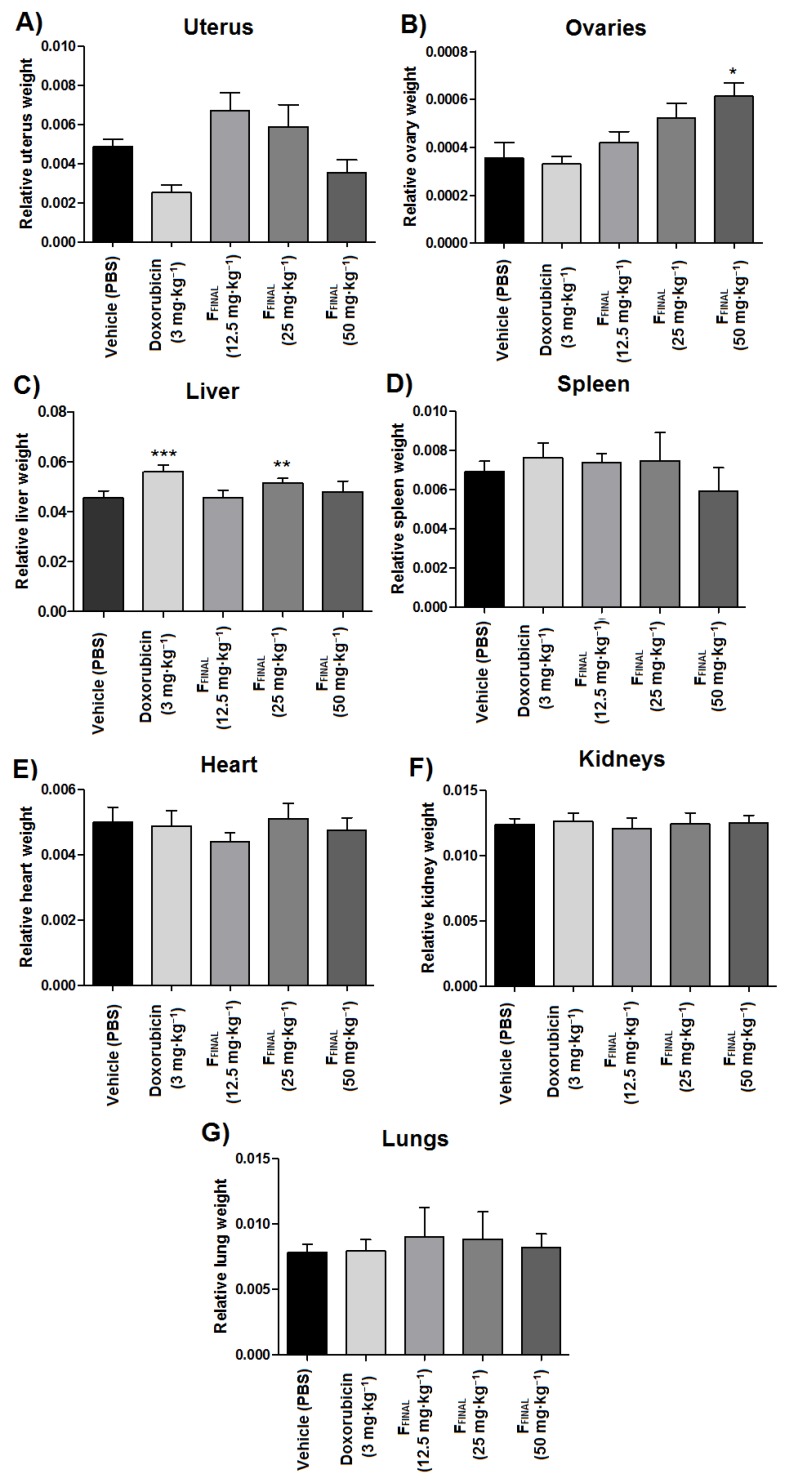
Relative weight of organs tested in the Ehrlich solid tumor model—F_FINAL_ fraction. Results expressed as mean ± standard deviation; Female Balb/c mice (*n* = 8 animals/group). (**A**) Uterus; (**B**) Ovaries; (**C**) Liver; (**D**) Spleen; (**E**) Heart; (**F**) Kidneys; (**G**) Lungs. Treatments (orally every day): vehicle (PBS pH 7.0 + Tween 80, 10 mL·kg^−1^), F_FINAL_ (12.5, 25 and 50 mg·kg^−1^); Treatments (i.p., every three days): Doxorubicin (3 mg·kg^−1^); * *p* < 0.05, ** *p* < 0.01 and *** *p* < 0.001 - different from vehicle group. Tukey test (ANOVA).

Some organs were weighed and macroscopically observed for possible signs of toxicity, such as weight gain (Figure 8). Comparing to vehicle group, livers from animals treated with doxorubicin and intermediate dose (25 mg·kg^−1^) of F_FINAL_ had a significant increase in relative weight (*p* < 0.001). Furthermore, the relative uterus weight of doxorubicin-treated animals decreased in comparison to the vehicle group (*p* < 0.05) while animals treated with the highest doses of F_FINAL_ showed an increase in the relative ovaries weight in comparison to the vehicle group (*p* < 0.001).

#### 3.3.3. Hollow Fiber Assays with MCF-7

Hollow fiber is a method used to propagate human cancer cells in inert fibers with small pores; enough to retain cancer cells but large enough to allow permeability of potential chemotherapeutic drugs [22]. As the human breast tumor cell line MCF-7 seemed to be the most sensible to F_FINAL_ fraction in in vitro anti-proliferative test, this cell line was selected to perform Hollow Fiber assay.

**Figure 9:**
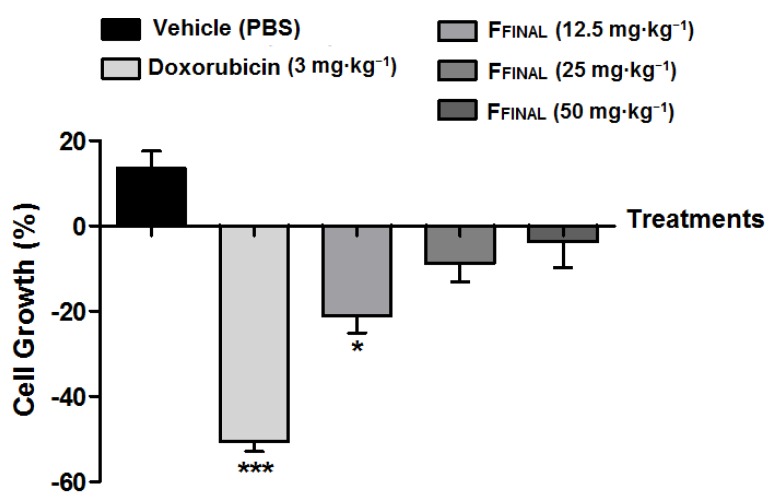
MCF7 cell proliferation, in Hollow fiber as a function of treatments. Results expressed as mean ± standard deviation; Female Balb/c mice (*n* = 5 animals/group). Treatments (orally every day): vehicle (PBS pH 7.0 + Tween 80, 10 mL·kg^−1^), F_FINAL_ (12.5, 25, and 50 mg·kg^−1^); Treatments (i.p., every three days): Doxorubicin (3 mg·kg^−1^). Statistical analysis by One-Way ANOVA, followed by Tukey’s test, * *p* < 0.05 and *** *p* < 0.001 related to vehicle group.

By comparing cell amounts inside the fibers at the beginning and at the end of the test, in the vehicle group (negative control) was observed cell proliferation of 13.5%. Doxorubicin treatment presented cytocidal activity of −50.5% as well as all doses of F_FINAL_ fraction (−21.1; −8.8 and −3.6% from lowest to highest concentration, respectively) (Figure 9). These results corroborate those obtained for the Ehrlich flank solid tumor experiment with the lowest dose (12.5 mg·kg^−1^) being the most efficient.

As expected by the results of Ehrlich solid tumor assay, doxorubicin treatment induced bodyweight loss at the 7th experimental day, as well as the F_FINAL_ fraction at 50 mg·kg^−1^ that promoted negative bodyweight variation at the 4th experimental day.

**Figure 10:**
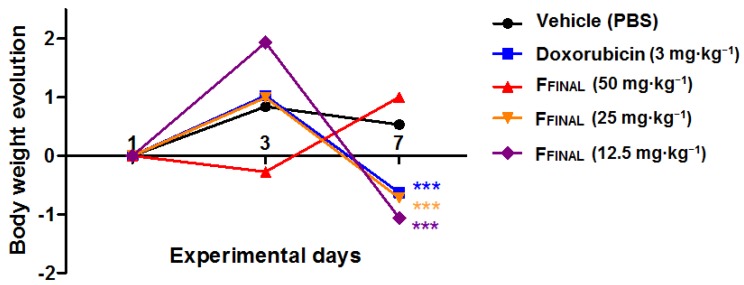
Animal bodyweight, variation during the Hollow fiber experiment—F_FINAL_ fraction. Bodyweight variation during 7 days of treatment; results expressed as mean of each experimental group; Female Balb/c mice (*n* = 5 animals/group); Treatments (orally every day): vehicle (PBS pH 7.0 + Tween 80, 10 mL·kg^−1^), F_FINAL_ (12.5, 25, and 50 mg·kg^−1^); Treatments (i.p., every three days): Doxorubicin (3 mg·kg^−1^). Statistical analysis by Kruskal–Wallis test followed by Dunn’s Multiple Comparison Test *** *p* < 0,001 compared to vehicle group.

Despite anti-proliferative effect on tumor cells for all tested concentrations, the F_FINAL_ fraction reduced animal weights at the highest dose at the beginning of treatments indicating animal bodyweight variation (Figure 10). The other doses presented toxic effect only at the end of the experiment. This toxicity has already been observed for the Ehrlich solid tumor test. The group treated with doxorubicin showed weight loss at the end of the experiment, which confirms collateral effects of this drug (bone marrow toxicity, gastrointestinal disorders, among others) that eventually limit its clinical use [33].

#### 3.3.4. Uterotrophic Activity in Prepubertal Rats

##### In vivo Anti-Estrogenic Activity of the F_FINAL_ Fraction

F_FINAL_ fraction was tested through the anti-estrogenic model in 21-day-old rats. The doses for this test were equal to the previous anticancer activity assay (50, 25, and 12.5 mg·kg^−1^).

**Figure 11:**
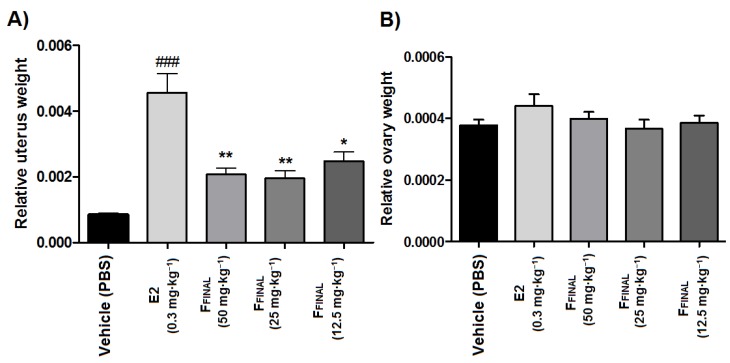
Relative weight of the (**A**) uterus (**B**) ovaries prepubertal Wistar rats after treatment with F_FINAL_ fraction in combination with 17-β-estradiol. Results expressed as mean ± standard deviation; Female Wistar mice (*n* = 8 animals/group); Treatments (orally, every three days): vehicle (PBS pH 7.0 + Tween 80, 10 mL·kg^−1^), F_FINAL_ (12.5, 25, and 50 mg·kg^−1^), 17β-estradiol (E2, 0.3 mg·kg^−1^), fifteen minutes before treatment with F_FINAL_; Tukey’s test (ANOVA).

As expected, the treatment with 17β-estradiol (E2, 0.3 mg·kg^−1^, orally) for three days significantly increased (*p* < 0.001) relative uterus weight of prepubertal rats when compared to the vehicle group, which is in agreement with the literature [25]. Differently, the treatment with F_FINAL_ (after 15 min of E2 administration), was able to significantly inhibit the proliferative effect of E2, regardless of F_FINAL_ concentration (Figure 11A). Furthermore, treatment with E2 and F_FINAL_ did not alter the relative weight of the ovaries of prepubertal rats (Figure 11B.) These results are in agreement with the observed profile of estrogenic/anti-estrogenic activity in vitro (E-screen), which F_FINAL_ fraction (1.25 and 2.5 μg·mL^−1^) was able to suppress the proliferative effect of 17β-estradiol on the MCF7 BUS cell line.

## 4. Discussion

This work investigated the anticancer and anti-estrogenic action of extract and fractions obtained from *Psidium guajava* L. Phytochemical analysis of F_FINAL_ fraction demonstrated 49% guajadial, that presented potent anti-proliferative activity on in vitro human tumor cell lines, with reduced TGI values, with selectivity for MCF-7 and MCF-7 BUS (5.59 and 2.27 μg·mL^−1^, respectively), suggesting a possible relationship between anti-proliferative activity and hormonal action. This correlation was confirmed both with E-screen and in vivo tests in uterus of prepubertal rats.

The choice of MCF7 BUS cell line to continue mechanism of action studies, considered the high sensitivity towards treatment with F_FINAL_ with a well-established estrogenic profile being sensitive to estradiol and therefore, most suitable for evaluating anti-estrogenic activity. Rizzo et al. [16] reported that guajadial enriched fraction presented structural similarities with estrogen and possible affinity to estrogenic receptors due to the chemical structure. The authors’ observed by in silico molecular docking model the potential hormonal activity of compounds obtained from *P. guajava* leaves.

Uboh et al. [34] reported that *P. guajava* extract leaves increased estradiol and progesterone levels in female rats, by 17.6 and 34.6% respectively. These observations indicated a great hormonal potential with possible action on estrogen receptors that prompted investigation in order to confirm such activity. Our data demonstrated, F_FINAL_ to significantly inhibit proliferative effect of estradiol on MCF7 BUS breast cells at the two highest concentrations tested (1.25 and 2.5 μg·mL^−1^), suggesting a straight relationship of effect with sample concentration.

Tumor cells that express estrogen receptors (ERs) indicate that cell proliferation depends on estrogen stimulation. Therefore, hormonal block may be a possible alternative to reduce cell growth. There are two alternatives to treatments available in this case: reduction of estrogens normally produced by the body and inhibition of connections between receptors and hormones. E-screen test result indicated that F_FINAL_ fraction presented compounds with a similar action to drugs which compete with estrogens in their receptor connection causing an inhibitory effect on cell proliferation. There is a valuable improvement in the process of drug development when the activity is associated with a specific receptor. The classification of receptors based on pharmacological responses remains a valuable and widely used approach [35].

One of the most striking features of cancer cells is the loss of cell division control, caused by genetic changes that alter this process. The main strategies of mechanisms used in cancer therapies development are reduction of tumor growth and induction of cell death [36]. The anti-proliferative activity test on BUS MCF-7 cell line was not satisfactory to establish mechanisms of cell death promoted by F_FINAL_ treatment. Therefore, evaluation of F_FINAL_ activity on cell cycle was performed by flow cytometry. F_FINAL_ stimulated a significant increase in G1 cell populations in both concentrations of 2.5 µg·mL^−1^ and 5.0 µg·mL^−1^, after 24 and 48 h, indicating possible impediment of cells to proceed to S phase. These results suggested that anticancer effect of guajadial enriched fraction does not involve exclusively the action on estrogenic receptors, but also cell death mechanism activation initiated by exposure of phosphatidylserine residues and interruption G1-phase cell cycle. The tamoxifen mechanism of action is well-established on cancer cells and consists in inhibiting DNA synthesis, inducing apoptosis and blocking cell cycle in G1 phase [37]. Similarly, F_FINAL_ fraction exhibited an activity on G1 phase of MCF-7 BUS cell cycle.

The treatment with F_FINAL_ after 15 min of estradiol (E2) administration was able to significantly inhibit proliferative effect on uterus, however, did not alter the relative weight of the prepubertal rats ovaries. This fact can be explained through the hormones involved in this process. Pituitary hormones FSH (follicle-stimulating hormone) and LH (luteinizing hormone) are related to ovarian stimulation and menstrual cycle. Hypothalamus regulates the release of pituitary hormones and is regulated by estrogen and progesterone (produced by the ovaries). Elevated levels of estrogens and progesterone stop the pituitary gland stimulation and therefore, blocking estrous cycle and consequently inhibiting ovulation. In prepubertal animals, this cycle is not yet active and, therefore, the ovary is not producing estrogens, which would influence the test results. The ovaries are mainly influenced by chorionic gonadotropin (produced by placenta), FSH and LH during prepubertal phase. These hormones, when injected into the prepubertal rat, will produce ovarian stimulation characterized by increased blood irrigation. In the test used, there would be changes in the ovaries only if the guava’s active ingredients had an effect on the FSH and LH ovarian receptors [38,39].

There are limitations related to cell culture such as predicting chemotherapy-response of solid tumors. Therefore, development of experimental models is crucial in order to enhance in vitro drug screening [40]. In this context, in vivo tests such as Ehrlich and Hollow fiber solid tumor models also confirmed the anticancer activity of *P. guajava* fraction evidenced by anti-proliferative activity in vitro. In this study, oral treatment with F_FINAL_ (12.5; 25 and 50 mg·kg^−1^) resulted in significant inhibition of solid tumors development and human tumor cells confined in semi-permeable fibers, as well as doxorubicin at a concentration of 3 mg·kg^−1^, demonstrating efficacy in solid tumors treatment. The effect produced by oral administration of extracts and fractions of *P. guajava* is highly relevant for the future development of a product, which could be an alternative to the chronic treatment carried out with tamoxifen and estrogenic antagonists.

## 5. Conclusions

The exploration of active compounds present in medicinal plants has become extremely essential in phytochemical studies associated with pharmacology. Guava leaves have been reported in the literature as promising natural source of anticancer agents. Results reported herein, using an enriched guajadial fraction, support the relevance of *Psidium guajava* L. as potential source of anticancer molecules. The enriched guajadial fraction exhibited promising anti-proliferative activity in vitro with selectivity for human breast cancer cell lines MCF-7 and MCF-7 BUS (TGI = 5.59 and 2.27 µg·mL^−1^, respectively). Furthermore, for the first time, anti-estrogenic activity in vivo of guajadial was investigated demonstrating that guajadial enriched fraction inhibited proliferative effect of estradiol on the uterus of pre-pubescent rats. These results indicate that guajadial acts in tumor inhibition through estrogen receptors, suggesting a tamoxifen-like mechanism of action. Therefore, *Psidium guajava* L. could be a promising source for development of new cancer treatments with further studies required to better understand cellular and molecular mechanisms of anti-cancer and anti-estrogenic action.

## Figures and Tables

**Table 1:** Human tumor and non-tumor cell lines used in in vitro assays.

	Cell Lines	Histology ^a^	D.I. ^h^ (10^4^ cell mL^−1^)
Tumor cell lines	U251 ^b^	CNS, Glioblastoma	4.0
	MCF 7 ^b^	Adenocarcinoma-mammary gland	6.0
	MCF7 BUS ^c^	Adenocarcinoma-mammary gland	6.0
	MDA-MB-231^d^	Adenocarcinoma-mammary gland	4.0
	NCI-ADR/RES ^b, e^	Adenocarcinoma-ovary	5.0
	OVCAR-3 ^b^	Adenocarcinoma-ovary	7.0
	786-0 ^b^	Adenocarcinoma-kidney	5.0
	NCI-H460 ^b^	Large Cell Carcinoma-lung	4.0
	PC-3 ^b^	Adenocarcinoma-prostate	4.5
	HT29 ^b^	Adenocarcinoma-colon	5.0
Immortalized cell lines	HaCaT ^f^	Spontaneously transformed keratinocytes from histologically normal skin	4.0
	MCF 10A ^g^	non-tumorigenic epithelial cells from mammary gland	4.0

^a^ Cell Miner (available at http://discover.nci.nih.gov/cellminer/), Cell line service (CLS) (available at http://www.clsgmbh.de/artikeldet.php?sprachnrs=2&sid=2bv11t49lt2e4s5msp8t7isho1&proid=800) and American Type Culture Collection (ATCC) (https://www.atcc.org/Products/All/CRL-10317.aspx#generalinformation); ^b^ donate by Frederick Cancer Research & Development Center, National Cancer Institute (NCI, EUA); ^c^ donate by UNESP/ Araraquara; ^d^ donate by USP/São Carlos; ^e^ multi-drug resistant cell line; ^f^ donate by FOP/UNICAMP; ^g^ donate by UNIFRAN; ^h^ D.I. = density of inoculation for anti-proliferative assay.

**Table 2:** Relative composition (%) of F_FINAL_ compounds.

Retention Time (RT) (min)	Relative Percentage (%)
28.08	50.7
45.01	6.4
46.71	42.9

**Table 3:** Anti-proliferative activity of F_FINAL_, dichloromethanic crude extract (DCE) and doxorubicin, against human cancer cell lines and one none tumor cell line.

	*TGI (µg·mL^−1^)		
	F_FINAL_	DCE	Doxorubicin
**U251**	3.90	61.5	1.22
**MCF-7**	5.59	64.61	11.39
**NCI/ADR-RES**	6.19	50.51	−0.12
**786-0**	7.69	62.88	4.20
**NCI-H460**	2.79	57.82	0.44
**PC-3**	3.70	39.94	1.52
**OVCAR-3**	2.66	27.23	2.12
**HT-29**	6.65	101.78	10.34
**HaCat**	2.91	58.39	0.35

*TGI: Total Growth Inhibition.

**Table 4:** Anti-proliferative activity of F_FINAL_ against four human breast cancer cell lines and one no tumor cell.

TGI (µg·mL^−1^)	
	F_FINAL_
**MCF-7**	8.86
**MCF-7 BUS**	2.27
**MDA MB 231**	5.13
**MCF 10A**	8.56

## Supplementary Materials

The following are available online: Figure S1. Representation of TLC showing fractions A, B and C obtained from DCE extract. Figure S2. Representation of TLC showing fractions A) B1, B2, B3 and B) B4 and B5 obtained from B fraction. Figure S3. Representation of TLC showing fractions A) B3.1, B3.2, B3.3 and B) B3.4 and B3.5 obtained from B3 fraction. C) Fractions were grouped by similarity and B3.2 fraction was named F_FINAL_. Figure S4. GC-MS analysis of F_FINAL_ fraction. (A) Chromatography profile and tandem mass spectrum of ion [M + H]^+^ *m/z* 474 at (B) 45.014 and (C) 46.706 min. Figure S5. Antiproliferative activity of the F_FINAL_ fraction (A), from dichloromethane crude extract of *Psidium guajava* L. Doxorrubicin (B) used as positive control. U251 (glioma), MCF-7 (breast), NCI-460 (lung, non-small cells), OVCAR-03 (ovarian), PC-3 (prostate), HT-29 (colon), 786-0 (renal), NCI-ADR/RES (ovarian expressing phenotype multiple drugs resistance), and HaCaT (human keratinocytes and immortalized nontumoral cells). Figure S6. Antiproliferative activity of the F_FINAL_ fraction, from dichloromethane crude extract of *Psidium guajava* L. MCF-7 BUS (estradiol receptor overexpressing mammary adenocarcinoma), MCF-10A (breast non-tumoral), MCF-7 (breast) and MDA MB 231.
