# Supplementary material for: Anti-Estrogenic Activity of Guajadial Fraction, from Guava Leaves (Psidium guajava L.)
Source: Molecules. 2020 Mar 27;25(7):1525. doi: 10.3390/molecules25071525 (PMC7181212; doi:10.3390/molecules25071525)
Supplement: Supplementary file 1 [file molecules-25-01525-s001.pdf]

Molecules

Antiestrogenic activity of Guajadial fraction, from Guava Leaves (*Psidium guajava* L.)

Jaqueline Moraes Bazioli [1,2], Jonas Henrique Costa [2], Larissa Shiozawa [1], Ana Lúcia Tasca Gois Ruiz[1], Mary Ann Foglio[1], João Ernesto de Carvalho[1,*]

[1] Faculty of Pharmaceutical Sciences, Universidade Estadual de Campinas, 13083-859 Campinas, SP, Brazil

[2] Institute of Chemistry, Universidade Estadual de Campinas, CP 6154, 13083-970 Campinas, SP, Brazil

*****carvalho@fcf.unicamp.br

Supplementary Material

2. Material and methods

2.5. Chromatographic analysis

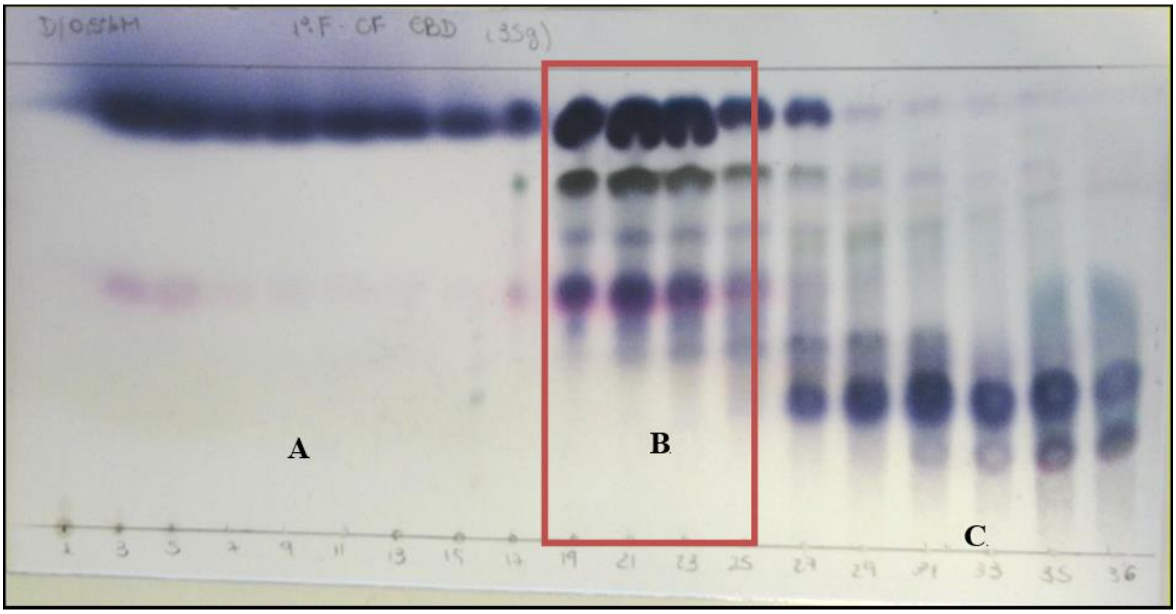

Figure S1. Representation of TLC showing fractions A, B and C obtained from DCE extract.

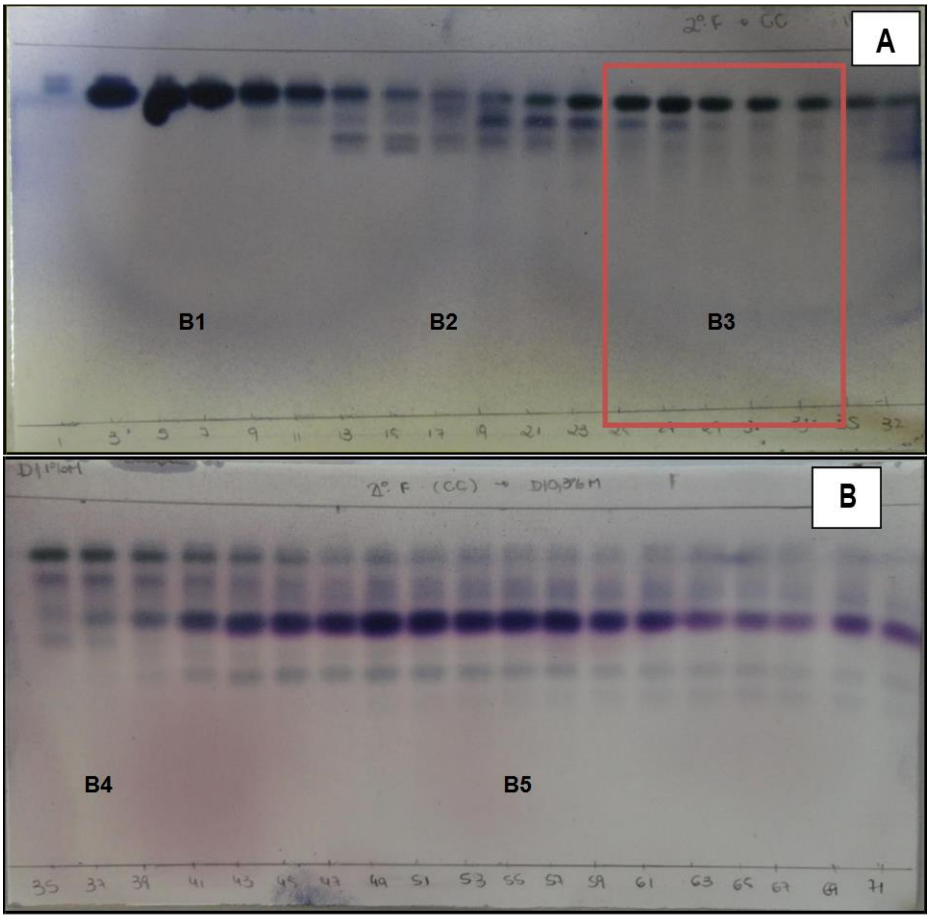

Figure S2. Representation of TLC showing fractions A) B1, B2, B3 and B) B4 and B5 obtained from B fraction.

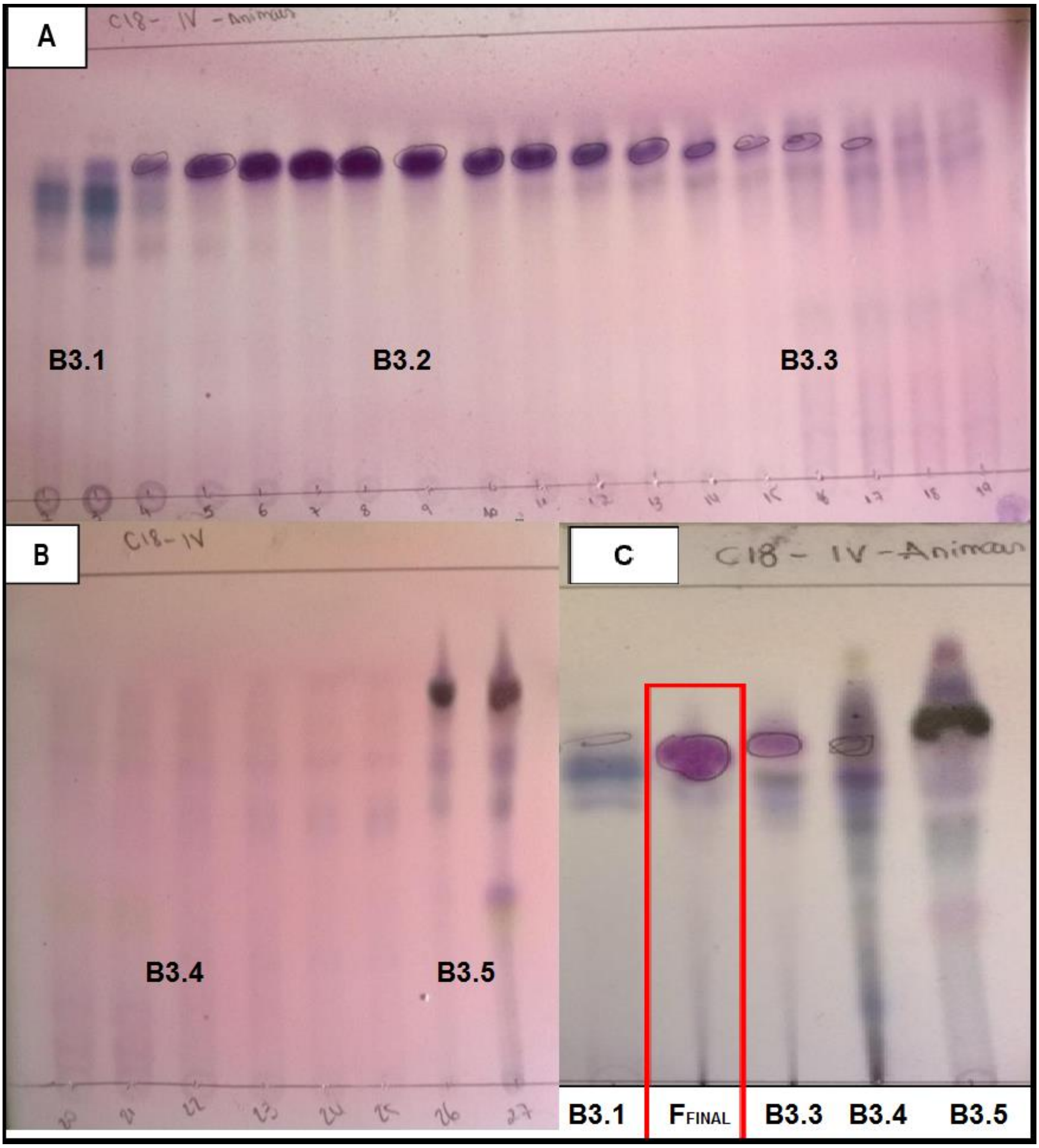

Figure S3. Representation of TLC showing fractions A) B3.1, B3.2, B3.3 and B) B3.4 and B3.5 obtained from B3 fraction. C) Fractions were grouped by similarity and B3.2 fraction was named F_{FINAL}.

3.1. Chromatographic analysis of F_{FINAL}

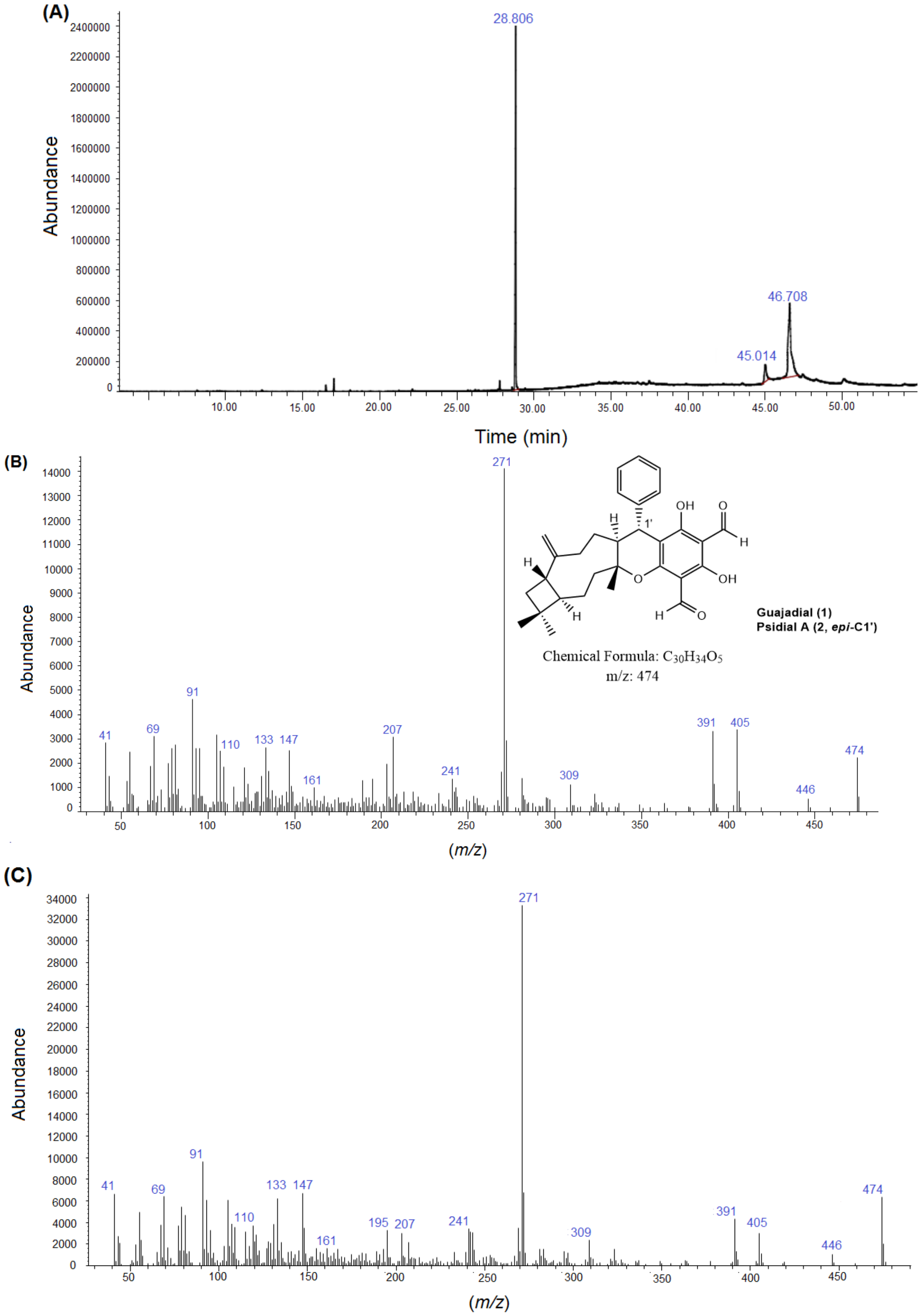

Figure S4. GC-MS analysis of F_{FINAL} fraction. (A) Chromatography profile and tandem mass spectrum of ion $[M+H]^+$ *m/z* 474 at (B) 45.014 and (C) 46.706 min.

3.2. In vitro assays

3.2.1. *In vitro* antiproliferative screening

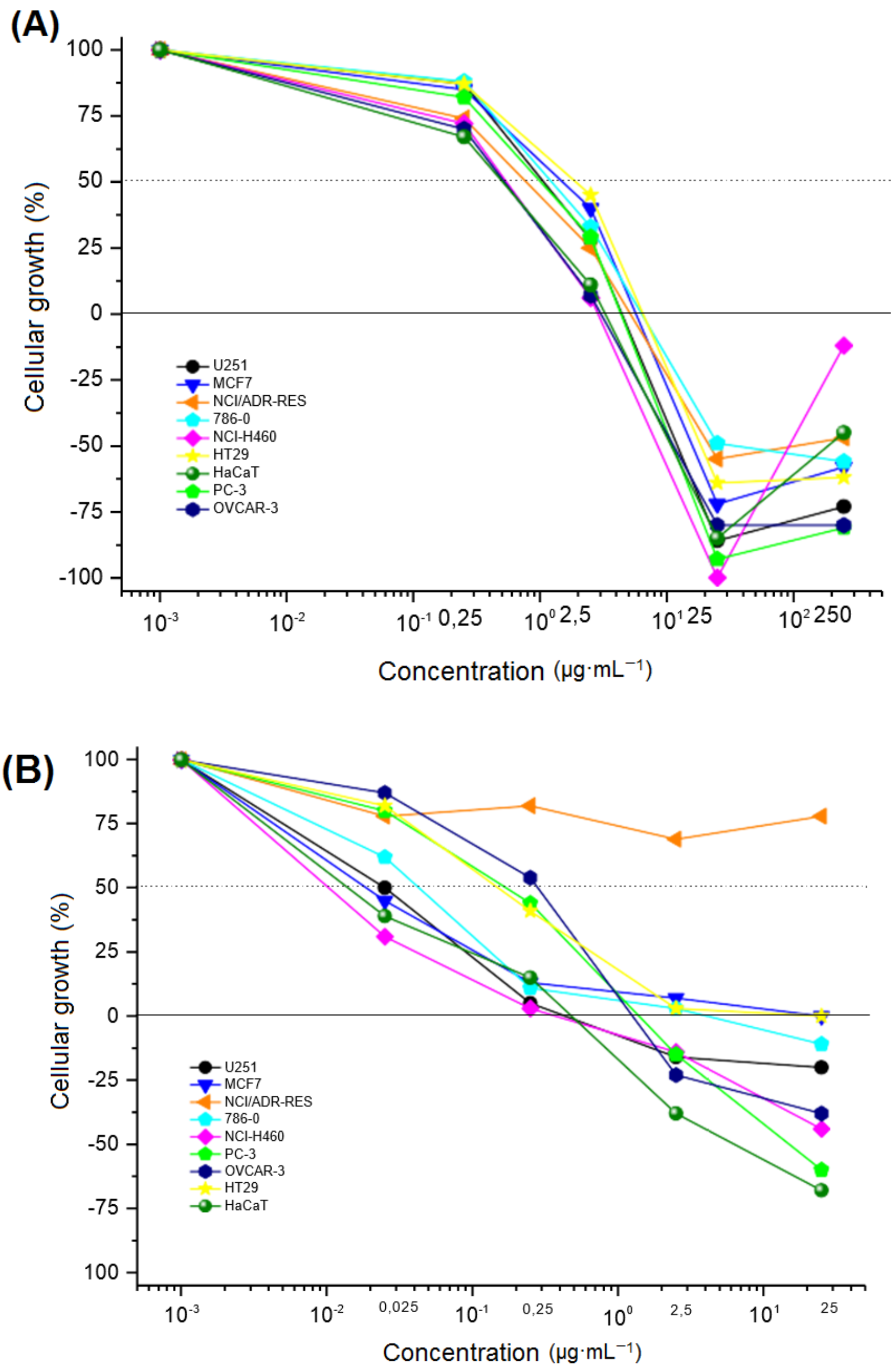

Figure S5. Antiproliferative activity of the F_{FINAL} fraction (A), from dichloromethane crude extract of *Psidium guajava* L. Doxorrubicin (B) used as positive control. U251 (glioma), MCF-7 (breast), NCI-460 (lung, non-small cells), OVCAR-03 (ovarian), PC-3 (prostate), HT-29 (colon), 786-0 (renal), NCI-ADR/RES (ovarian expressing phenotype multiple drugs resistance), and HaCaT (human keratinocytes and immortalized nontumoral cells).

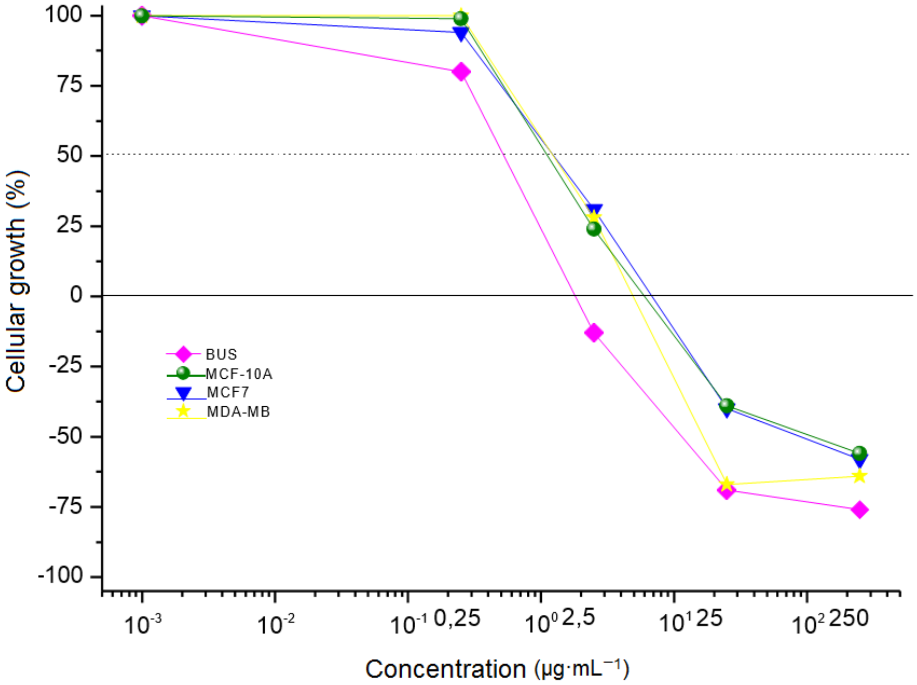

Figure S6. Antiproliferative activity of the F_{FINAL} fraction, from dichloromethane crude extract of *Psidium guajava* L. MCF-7 BUS (estradiol receptor overexpressing mammary adenocarcinoma), MCF-10A (breast non-tumoral), MCF-7 (breast) and MDA MB 231.
